# Reanalysis and External Validation of a Decision Tree Model for Detecting Unrecognized Diabetes in Rural Chinese Individuals

**DOI:** 10.1155/2017/3894870

**Published:** 2017-05-30

**Authors:** Zhong Xin, Lin Hua, Xu-Hong Wang, Dong Zhao, Cai-Guo Yu, Ya-Hong Ma, Lei Zhao, Xi Cao, Jin-Kui Yang

**Affiliations:** ^1^Department of Endocrinology, Beijing Tongren Hospital, Capital Medical University, 1 Dong Jiao Min Xiang, Beijing 100730, China; ^2^Beijing Key Laboratory of Diabetes Prevention and Research, 1 Dong Jiao Min Xiang, Beijing 100730, China; ^3^Department of Mathematics, School of Biomedical Engineering, Capital Medical University, Beijing 100069, China; ^4^Department of Endocrinology, Beijing Luhe Hospital, Capital Medical University, Beijing 101148, China

## Abstract

We reanalyzed previous data to develop a more simplified decision tree model as a screening tool for unrecognized diabetes, using basic information in Beijing community health records. Then, the model was validated in another rural town. Only three non-laboratory-based risk factors (age, BMI, and presence of hypertension) with fewer branches were used in the new model. The sensitivity, specificity, positive predictive value, negative predictive value, and area under the curve (AUC) for detecting diabetes were calculated. The AUC values in internal and external validation groups were 0.708 and 0.629, respectively. Subjects with high risk of diabetes had significantly higher HOMA-IR, but no significant difference in HOMA-B was observed. This simple tool will help general practitioners and residents assess the risk of diabetes quickly and easily. This study also validates the strong associations of insulin resistance and early stage of diabetes, suggesting that more attention should be paid to the current model in rural Chinese adult populations.

## 1. Introduction

The prevalence of diabetes is increasing, constituting a serious public health problem in China [1, 2]. The cost of treating diabetes and related complications is also a major concern [3]. A national study showed that 9.7% of the adult population has diabetes, according to oral glucose tolerance test (OGTT) results; meanwhile, diabetes is undiagnosed in 60.7% of these cases [1]. A large proportion of diabetes cases are undetected, with many presenting diabetic complications at the time of clinical diagnosis in rural China [4]. However, OGTT cannot be performed for detecting diabetes in all adults because of large population and limited health resources.

Diabetes screening in community populations is important as clinical symptoms of undiagnosed type 2 diabetes (T2DM) patients are not specific. Some methods have been developed to screen individuals at high risk of undiagnosed diabetes [5, 6]. However, their applicability and simplicity for the Chinese population is uncertain [7–9]. In 2010, we used the decision tree method to develop a screening tool for diabetes and prediabetes utilizing common health information [10]. At present, to develop community health service, the Beijing municipal government requires urban and rural residents to establish electronic community health records. Therefore, using community health records information to screen high-risk subjects for diabetes has become possible. In our previous study for detecting diabetes, five risk factors, including age, waist/hip ratio (WHR), waist, duration of hypertension, and weight, were included. However, special risk factors, such as duration of hypertension, are not mentioned in community health records, while basic information such as age, BMI, and blood pressure situations are included. Thus, we realized that the above screening tool should be simplified further.

In this study, we reanalyzed our previous data and developed a new decision tree model using simple and basic parameters included in community health records. We also externally validated this simple tool in another rural population.

## 2. Materials and Methods

### 2.1. Ethics Statement

This study was conducted with approval from the Ethics Committee of Beijing Tongren Hospital, Capital Medical University. Written informed consent was obtained from each participant.

### 2.2. Study Population

Two different populations were used in this study. Population 1 was used to develop the model and population 2 for validation.

Population 1 was selected from the Beijing Community Pre-Diabetes (BCPD) study conducted in the year 2007 in Nanfaxin, a satellite rural town of Beijing [10, 11]. Participants without previously known diabetes were selected from the 2826 registered individuals aged ≥ 35 years. The subjects were invited to undergo baseline examinations, including anthropometric and blood pressure measurements, in addition to completing a general health questionnaire. Subjects with fasting plasma glucose (FPG) ≥ 5.6 mmol/L were submitted to an OGTT [12]. According to the 1999 World Health Organization criteria [1], a total of 220 new cases of diabetes and 1868 subjects with normal blood glucose were identified. Then, we built a decision tree model that can divide the subjects into “high risk” (“high risk 1”) and “low risk” (“low risk 1”) groups of diabetes.

**Figure 1:**
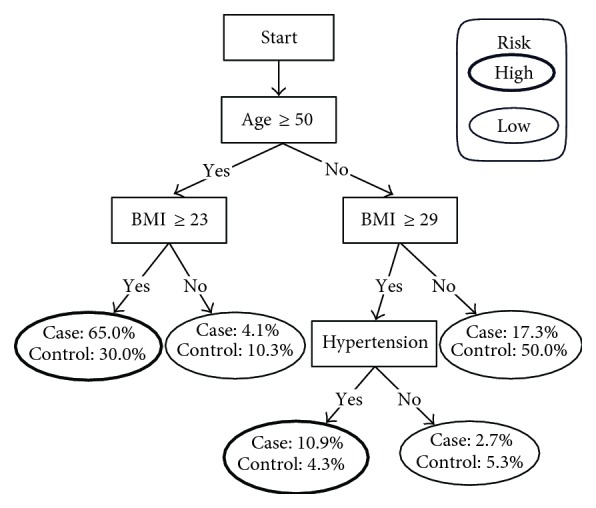
Simple decision tree for detecting diabetes in population 1. Hypertension: SBP ≥ 140 mmHg and/or DBP ≥ 90 mmHg or self-reported history of hypertension.

Population 2 was selected from a cross-sectional, population-based study—the Diabetes and Other Chronic Disease Survey, conducted in Mizidian, another rural satellite town of Beijing, with 28 villages and a permanent resident population of 23,000. Between 2013 and 2014, 3300 men and women aged ≥ 20 years were randomly selected to participate in the study by the Center for Disease Control and Prevention (CDC) of Tongzhou District, Beijing, and local general practices. All subjects underwent baseline examinations, including anthropometric and blood pressure measurements, and completed a general health questionnaire obtained through face-to-face inquiry, with questions on personal and family history of disease, medication, and lifestyle factors. Participants were also asked whether a doctor had ever told them that they had any of the conditions contained in a list that included diabetes and hypertension. After excluding individuals who suffered from diabetes, a total of 2967 individuals without a history of diabetes were divided into “high risk” (“high risk 2”) and “low risk” (“low risk 2”) groups. In this population, “high risk 2” was defined as (1) previous fasting blood glucose (FBG) levels above 5.6 mmol/l or (2) classification as “high risk 1” according to the diabetes decision tree model (Figure 1). Of the 2967 individuals, 1844 were “high risk,” and 1123 “low risk.” Because the main application of the decision tree model is to screen high-risk subjects, we relatively reduced the screening number in low-risk subjects. We randomly selected one-half of “high risk” subjects and one-third of “low risk” individuals. Finally, a total of 910 (98.7%) “high risk” and 299 (79.9%) “low risk” subjects underwent OGTT, HbA1c, renal and liver function, blood lipid, and fasting insulin measurements.

### 2.3. Laboratory Measurements

Blood samples were collected after overnight fasting for the determination of plasma glucose and HbA1c levels. Blood specimens were collected after fasting; the OGTT was performed between 08:00 and 10:00 hours. These specimens were analyzed within 24 hours. Plasma glucose was determined by the glucose oxidize method, and HbA1c was measured by high-performance liquid chromatography (VARIANT, Bio-Rad Lab., Hercules, CA, USA). Serum insulin levels were measured with ADVIA Centaur Immunoassay System (Siemens Medical Solutions Diagnostics). Insulin resistance (IR) and beta-cell secretion function were calculated using the homeostasis model by Matthews et al. as follows: fasting serum insulin (FINS, *μ*U/ml) × fasting serum glucose (FPG, mmol/l)/22.5 (HOMA-IR) and 20 × FINS/(FPG-3.5) (HOMA-B).

### 2.4. Statistical Analysis

We built a model using the decision tree method. A decision tree is a nonlinear discrimination method, which can split a sample into progressively smaller subgroups. The procedure selects the independent variable that has the strongest association with the dependent variable [13]. The decision rules provide specific information about risk factors based on rule induction. In our analysis, the target variable was whether diabetes is present or absent. Starting at the tree root, data were split into two groups that best separated the target classes. The process was then repeated for each of the child nodes until all the subjects were assigned to “high risk” or “low risk” group. As the number of cases of undiagnosed diabetes in population 1 was small, in order to derive a reliable conclusion, a decision was made to assess a number-matching sample. Three hundred subjects were randomly selected from all normal controls (*n* = 1868), and all diabetes subjects were used to develop a model [14, 15]. Because the vast majority of individuals in rural China are unwilling to undergo blood tests with no disease history, the data utilized in this tool were noninvasive. The risk factors included in the classification tree model were age, gender, body mass index (BMI), presence of hypertension, and family history of diabetes. The “leave-one-out” method [16] was used to verify our conclusions. Population 2 was used to externally validate the model.

To test accuracy, we calculated the sensitivity, specificity, positive predictive value, and negative predictive value; receiver operating characteristic (ROC) curves were plotted; and area under the curve (AUC) values were obtained for the two populations. Statistical analyses were performed with “SPSS version 11.5” (SPSS Inc., Chicago, IL, USA) for Windows and the “R software” (http://www.r-project.org). Two-sided *p* < 0.05 was considered statistically significant.

## 3. Results

Table 1 shows the characteristics of population 1. Patients with diabetes or prediabetes had significantly increased age, waist circumference, BMI, systolic pressure, and diastolic pressure, compared with those with normal blood glucose. They were also significantly more likely to have a family history of diabetes and a history of hypertension.

**Figure 2:**
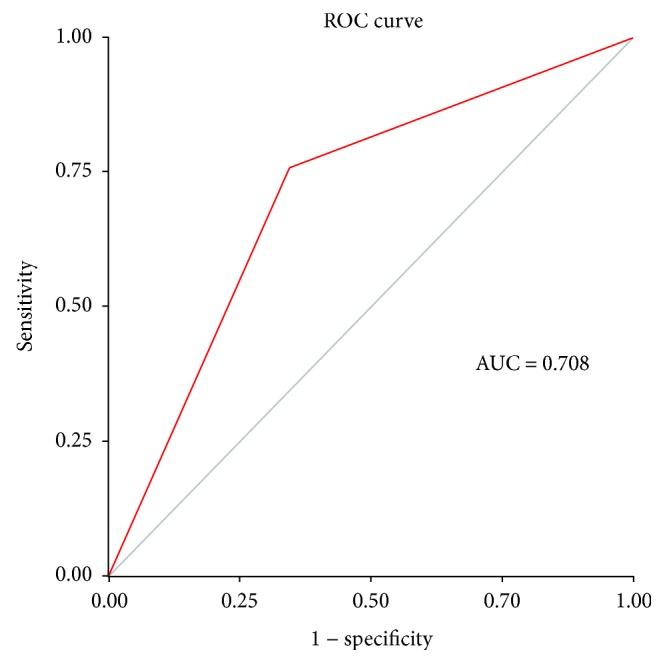
ROC curve of simple decision tree for detecting diabetes in population 1.

The decision tree illustrates decision rules (Figure 1). Among the clinical parameters analyzed, three factors were selected by the program for the decision tree, including age, BMI, and the presence of hypertension. As shown in Figure 1, patients over 50 years old with BMI above 23 kg/m^2^ were at high risk for diabetes; those below 50 years old with BMI above 29 kg/m^2^ and hypertension were also at high risk for diabetes. Subjects in the “high risk” group need to be diagnosed as soon as possible. Sensitivity, specificity, positive predictive value, negative predictive value, and AUC for diabetes detection were 0.759 (0.697–0.814), 0.657 (0.600–0.710), 0.618 (0.558–0.677), 0.788 (0.732–0.837), and 0.708 (0.663–0.753), respectively (Figure 2).

**Figure 3:**
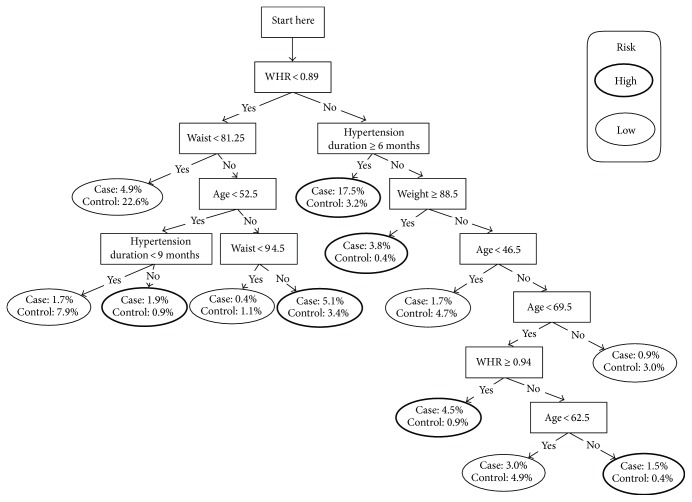
Decision tree for detecting diabetes in population 1 (2010). WHR, waist/hip ratio.

To examine the differences in detection power between the previous relatively complex model (Figure 3) [10] and the current simple one (Figure 1), we compared their AUC values. The general approach to assessing the difference in areas under the two ROC curves is to use generalized Z-statistics [11]. No significant difference was found between the two models (*z* = 1.91, *p* = 0.056).

**Figure 4:**
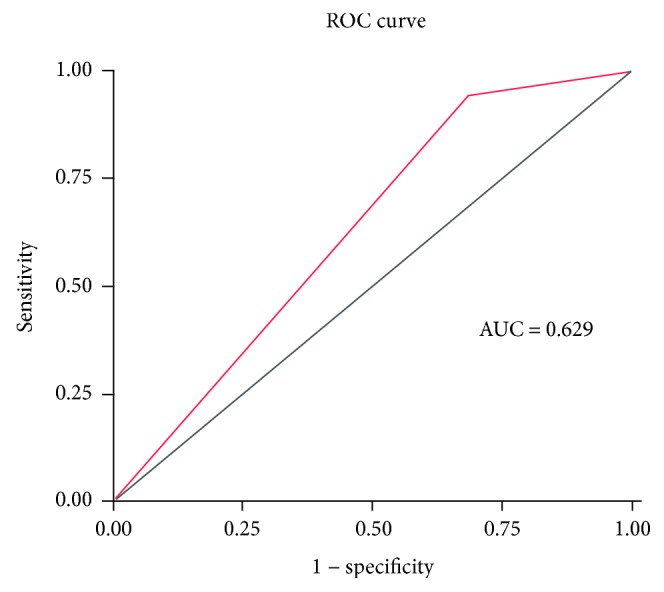
ROC curve of simple decision tree for detecting diabetes in population 2.

Table 2 shows the characteristics of population 2. High-risk subjects showed significantly increased age, waist circumference, BMI, and blood pressure compared with the low-risk group. High-risk subjects also had higher fasting plasma glucose (FPG), 2 h post oral glucose load plasma glucose (2-hPG), HbA1c, cholesterol (CHO), triglyceride (TG), and low-density lipoprotein cholesterol (LDL-C) levels. We also assessed FINS levels and calculated HOMA-IR and HOMA-B values. High-risk subjects had significantly higher HOMA-IR compared with the low-risk group, but showed no significant difference in HOMA-B. Additionally, there were significantly more diabetes and prediabetes subjects in the high-risk group. Sensitivity, specificity, positive predictive value, negative predictive value, and AUC for diabetes detection were 0.943 (0.886–0.977), 0.316 (0.284–0.348), 0.171 (0.143–0.201), 0.974 (0.947–0.989), and 0.629 (0.584–0.67), respectively (Figure 4()).

To assess the validity of our findings, we calculated the sample size of ROC curve analysis using the “pROC package” of the “R software” (http://www.r-project.org). With a high-risk population of 910 cases, a low-risk population of 299 cases, and an AUC of 0.629 (decision tree for detecting diabetes), the statistical power of the test was close to 100.0%. Therefore, the sample size used for validating the decision tree model was sufficient.

## 4. Discussion

Because of rapidly increasing diabetes prevalence and the threat of diabetic complications, identifying individuals with undiagnosed T2DM is important for both clinical practice and public health. According to current diagnostic criteria, the diagnosis of diabetes relies heavily on OGTT. If all subjects are submitted to standard procedures for blood glucose determination, a great deal of resources would be consumed with low efficiency, and unnecessary individual screening required. Therefore, screening individuals at high risk is recommended [5, 6, 17]. Screening methods of diabetes have been developed, but their applicability to different populations is uncertain [18, 19]. In this study, we developed and externally validated a simple tool that can be used to screen undiagnosed diabetes in rural Chinese areas. Because this study aimed to popularize the use of basic information in community health records, only three simple factors, that is, age, BMI, and presence of hypertension, are required in the decision tree model. Additionally, the model showed moderate detection accuracy with no significant difference compared with the relatively complex counterpart described previously [10]. As community residents' health record system in Beijing is increasingly comprehensive, using it to screen high-risk subjects for diabetes has become possible. Not only practitioners but also community residents can easily determine the risk of diabetes, according to the decision tree model using the above three basic factors.

Normally, the more risk factors and higher complexity a screening method has, the better its performance. Unfortunately, their applicability may be less efficient. For example, some studies are in the form of formulas, and conclusions cannot be drawn immediately [10]. The New Chinese Diabetes Risk Score includes age, sex, waist circumference, BMI, systolic blood pressure, and family history of diabetes (six risk factors), with an AUC in external validation populations of 0.725 [9]. Our previous decision tree model for detecting diabetes comprises five risk factors, including age, waist/hip ratio (WHR), waist, duration of hypertension and weight, for an AUC of 0.731. In decision tree methods, conclusion can be obtained from the root to terminal nodes through some branches [10]. In this study, although only three very simple non-laboratory-based factors with fewer branches were used, AUC values in internal and external validation groups were 0.708 and 0.629, respectively. The relative risk of diabetes in high-risk subjects was 6.507 times higher than that in the low-risk group for the external validation population. This finding demonstrated that the model is very efficient for screening diabetes.

To further assess the high- and low-risk groups, additional examinations were performed, including anthropometric, blood pressure measurements, and laboratory tests, in population 2. Interestingly, high-risk subjects had not only higher blood glucose and HbA1c levels but also increased CHO, TG, and LDL-C levels. Furthermore, high-risk subjects had higher fasting insulin and elevated HOMA-IR levels. The mechanism of T2DM includes insulin resistance and insulin deficiency. In population 2, no significant difference in HOMA-B was observed between high- and low-risk subjects. This demonstrates that individuals at high risk of diabetes have a significant insulin resistance state. This evidence supports the strong relationship between insulin resistance and the early stage of diabetes. It also demonstrates that insulin resistance may be an earlier starting mechanism in Chinese individuals.

Limitations of this study should be mentioned. First, we calculated the kappa coefficient of the current model; although we could not obtain a good kappa coefficient, the model displayed high sensitivity (0.943, 95% CI: 0.886–0.977). The aim of this study was to detect unrecognized diabetes as much as possible in the general population; therefore, sensitivity was the focus index. Second, the prevalence of diabetes was relatively high in our study population, which may be explained by the site of subject selection. The development and validation of the model was based on the information provided by only two regions; thus, our results may be validated with more available samples.

In conclusion, we developed and validated a very simple tool that can be used to screen undiagnosed diabetics in rural Chinese populations. This will help general practitioners and residents assess the risk of diabetes quickly and easily. This study also validates the strong associations of insulin resistance and early stage of diabetes, suggesting that more attention should be paid to the current model in rural Chinese adult populations.

## Figures and Tables

**Table 1:** Characteristics of population 1.

	Normal	Prediabetes	Diabetes
Number	1868	173	220
Sex (M/F)	638/1230	71/102	108/112
Age (year)	51.66 ± 10.35	56.15 ± 9.69^∗^	56.22 ± 10.06^∗^
Body mass index (kg/m^2^)	25.72 ± 3.74	27.00 ± 3.79^∗^	27.91 ± 3.93^∗^
Waist circumference (cm)	85.45 ± 10.18	89.90 ± 10.29^∗^	93.59 ± 10.22^∗^
Systolic pressure (mmHg)	131.57 ± 18.61	138.16 ± 17.76^∗^	137.75 ± 16.40^∗^
Diastolic pressure (mmHg)	81.27 ± 11.42	84.76 ± 17.76^∗^	83.56 ± 11.09^∗^
% with a family history of diabetes^a^	3.40%	11.00%^∗^	11.40%^∗^
% with history of hypertension	17.70%	43.90%^∗^	47.30%^∗^

Values are means (SD) or *n* (%). ^a^Either a sibling or parent with diabetes; compared to the normal subjects by unpaired *t*-test or Chi-square test; ^∗^versus normal *P* < 0.001.

**Table 2:** Characteristics of population 2.

	All	Low risk	High risk
Number	1209	299	910
Sex (M/F)	415/794	103/196	312/598
Age (year)	55.84 ± 6.91	50.41 ± 8.52	57.62 ± 5.18^∗^
Diabetes	123 (10.17%)	7 (2.34%)	116 (12.75%)^∗^
Prediabetes	262 (21.67%)	32 (10.70%)	230 (25.27%)^∗^
Body mass index (kg/m^2^)	26.58 ± 3.61	24.00 ± 3.30	27.43 ± 3.30^∗^
Waist circumference (cm)	92.60 ± 9.92	86.16 ± 9.25	94.71 ± 9.20^∗^
Systolic pressure	132.88 ± 18.63	125.68 ± 16.61	135.25 ± 18.66^∗^
Diastolic pressure	78.21 ± 10.96	75.87 ± 10.81	78.97 ± 10.90^∗^
% with a family history of diabetes^a^	8.68%	10.00%	8.20%
% with history of hypertension	36.5%	20.10%	41.90%^∗^
FPG (mmol/l)	5.72 ± 0.98	5.42 ± 0.95	5.82 ± 0.97^∗^
2hPG (mmol/l)	6.88 ± 2.80	5.85 ± 1.61	7.22 ± 3.02^∗^
HbA1c (%)	5.89 ± 0.58	5.67 ± 0.34	5.96 ± 0.63^∗^
Fasting insulin (*μ*U/ml)	47.16 (29.92, 68.59)	39.22 (26.63, 61.99)	49.16 (32.73, 70.50)^∗∗^
HOMA-IR	10.64 (6.68, 15.74)	8.33 (5.64, 13.53)	11.24 (7.43, 16.48)^∗^
HOMA-B	30.99 (19.89, 46.04)	30.99 (20.07, 50.12)	31.01 (19.83, 45.09)
CHO (mmol/l)	5.06 ± 0.96	4.83 ± 0.85	5.13 ± 0.98^∗^
TG (mmol/l)	1.47 (0.99, 2.16)	1.23 (0.88, 1.82)	1.54 (1.05, 2.24)^∗^
HDL (mmol/l)	1.36 ± 0.43	1.38 ± 0.35	1.35 ± 0.46
LDL (mmol/l)	3.07 ± 0.80	2.89 ± 0.72	3.13 ± 0.81^∗^

Values are means (SD), median (range), or *n* (%). ^a^Either a sibling or parent with diabetes; comparison between low-risk and high-risk groups by unpaired *t*-test or Chi-square test; ^∗^versus normal *P* < 0.001; ^∗∗^versus normal *P* < 0.01.
